# Understanding valuation of travel time changes: are preferences different under different stated choice design settings?

**DOI:** 10.1007/s11116-016-9716-4

**Published:** 2016-06-23

**Authors:** Manuel Ojeda-Cabral, Stephane Hess, Richard Batley

**Affiliations:** Institute for Transport Studies, University of Leeds, 30-40 University Road, Leeds, LS2 9JT UK

**Keywords:** Stated choice, Experimental design, Value of travel time, Cost damping, Small time savings, Design effects

## Abstract

Stated choice (SC) experiments are the most popular method to estimate the value of travel time changes (VTTC) of a population. In the simplest VTTC experiment, the SC design variables are time changes and cost changes. The levels of these variables create a particular setting from which preferences are inferred. This paper tries to answer the question “do preferences vary with SC settings?”. For this, we investigate the role of the variables used in the SC experiment on the estimation of the set of VTTC (i.e. mean and covariates). Ideally, one would like to observe the same individuals completing different SC experiments. Since that option is not available, an alternative approach is to use a large dataset of responses, and split it according to different levels of the variable of interest. We refer to this as partial data analysis. The estimation of the same model on each sub-sample provides insights into potential effects of the variable of interest. This approach is applied in relation to three design variables on the data for the last national VTTC study in the UK, using state-of-the-art model specifications. The results show several ways in which the estimated set of VTTC can be affected by the levels of SC design variables. We conclude that model estimates (including the VTTC and covariates) are different in different settings. Hence by focussing the survey on specific settings, sample level results will be affected accordingly. Our findings have implications for appraisal and can inform the construction of future SC experiments.

## Introduction

The value of travel time is a key element in the appraisal of transport projects (Small 2012; Börjesson and Eliasson 2014). Big national studies are carried out at somewhat regular intervals to obtain an estimate of how the population would value changes in travel time. The objective is therefore a measure of the value of travel time changes (VTTC). Since valuation is likely to vary across the population and with the trip context, the interest is not placed on obtaining a single VTTC but an index according to some variables that could explain travellers’ VTTC. Among these variables are individual characteristics (e.g. income) and trip characteristics (e.g. journey length). The recommended set of VTTC for appraisal has significant economic implications, and it crucially affects which projects are carried out in a country. Small (2012) and Daly et al. (2014) concur that our understanding of the VTTC and its variation should increase in order to maintain the credibility of the VTTC concept. Hence, it is important to pursue both an unbiased estimate of the VTTC and unbiased estimates of VTTC elasticities with respect to the variables of interest.

**Fig. 1:**
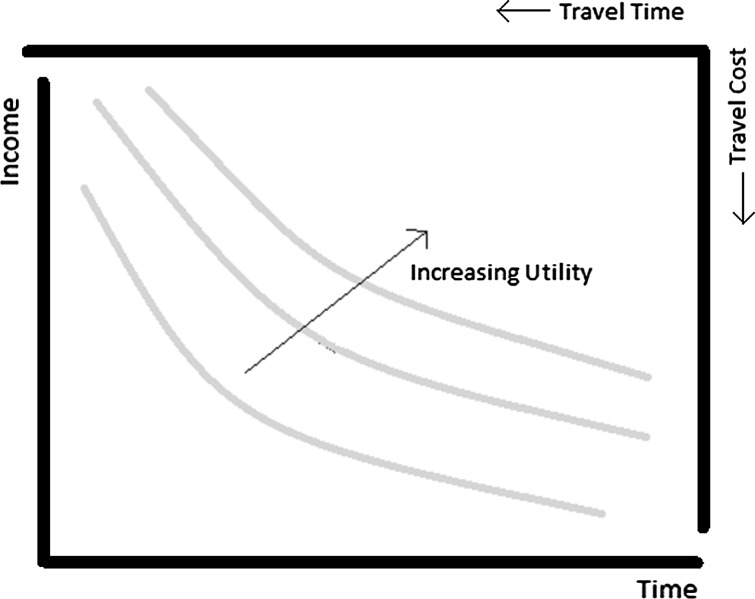
Indifference curves for the money-time trade-off

The theory of the VTTC has been well rehearsed over the last five decades, based mainly on the seminal work on time allocation by Becker (1965). DeSerpa (1971) developed a framework, building upon Becker’s work, from which a theoretical definition of the VTTC is obtained. In short, the VTTC is defined as the marginal rate of substitution between travel time and money (e.g. travel cost). It is then widely agreed that the VTTC can be found in individuals’ travel choices where they trade-off money and time. Simply imagine a classical convex indifference curve for the consumption of two goods, time and money, that can be changed by the allocation of time and money to travelling (as the Fig. 1 below depicts).

In this framework, utility increases as the individuals reduce their allocation of time and income to travelling and the slope of each indifference curve represents the VTTC. While some data of this nature exist from real travel markets (i.e. revealed preference data), it is often very limited: individuals’ travel choices are observable, but it is hard to observe the actual trade-off (if any) that the person faced at the moment of choice. For this reason, experiments that mimic those necessary trade-offs and choices are typically conducted to collect data. National VTTC studies typically rely on hypothetical stated choice (SC) experiments for data collection. Through these methods the researcher has more control over the variables of interest, time and cost, and can generate trade-offs. Discrete choice models grounded in traditional microeconomic theory are then employed to estimate the VTTC from the data. In this context, the artificial nature of the SC experiments has sometimes generated doubts on the validity of estimation results (e.g. Daly and Tsang 2009). For example, Ojeda-Cabral et al. (2015) found surprisingly similar patterns in the variation of the VTTC with income and journey length in two different countries (UK and Denmark) which had used the same SC design. While there is plenty of evidence on how modelling specification can also affect the estimation results (see also Fosgerau et al. 2007a, b; and Fosgerau and Bierlaire 2009), we are not aware of any work that shows how the preferences of individuals vary (reflected by model estimates) across a number of different SC design settings.

Our global aim is to increase our understanding of the set of VTTC in a population. In this paper we do not question the validity of VTTC estimation results using SC experiments. However, we acknowledge that the variables used in the SC experiments (decided by the researcher^1^) may influence the estimated set of VTTC, because preferences may be different under different SC settings. There is a subtle difference here from saying that the survey influences the behaviour; rather, different survey settings will potentially lead to different results given that preferences may vary across settings.

In the simplest VTTC experiment, the design variables are travel time changes (∆t) and travel cost changes (∆c). The ratio ∆c/∆t forms a valuation threshold (Boundary VTTC, or BVTTC). It is essential to understand how SC design variables influence the VTTC and other estimates in order to improve the construction of SC experiments: if preferences do vary with the setting, researchers should decide for which particular setting/s they want to infer preferences.

The objective of the paper is therefore to investigate how different (or not) the preferences of a sample of travellers may be under different SC design settings. Preferences are represented by the model estimates of the underlying set of VTTC, while the SC design settings are represented by the levels of the SC design variables. For this, a series of empirical exercises which we refer to as partial data analysis are conducted. Ideally, one would like to observe the same individuals completing different SC experiments. However, with the data currently available, an alternative approach is to use a large dataset of individuals’ responses, and split it according to different levels of the variable of interest. The data can then be analysed *as if* the design had a restricted range for the variable of interest (e.g. Fosgerau and Börjesson 2015), where it is still highly likely by design that a given respondent contributes to the results in different segments, given the variety in the settings they are faced with.

To increase the policy relevance of this work, we use the data for the last published national VTTC study in the UK (Mackie et al. 2003) and state-of-the-art model specifications. The data will be split based on the levels of the three design variables: boundary VTTC, ∆t and ∆c. The estimation of the same advanced model on each sub-sample will show preferences for a particular setting (defined by the levels of the variable). This paper contributes to the literature on the estimation of the value of time by showing how preferences may (or not) be different under different SC designs, which is important both for researchers and policy-makers. The VTTC and how it varies across and within people remain not fully understood, and not enough attention has been paid to the SC designs that define the choice contexts (e.g. Fosgerau and Börjesson 2015). Our work will increase our understanding, will be useful to inform the construction of future SC experiments to estimate valuations of travel time and will have implications for appraisal.

The paper is structured as follows. ”Evidence on variation in the VTTC”section presents some existing evidence on how the VTTC has been found to vary across and within individuals, which shapes the current perceptions about the set of VTTC in a population. “Methodology to estimate the VTTC” section reviews the standard methodology applied in most European studies to estimate the VTTC, explaining the essence of SC experiments and the most common discrete choice models in the field. “Dataset” section introduces the dataset and “Empirical work” section describes the empirical work, including all results. “Discussion and conclusions” section concludes.

## Evidence on variation in the VTTC

Empirical evidence strongly suggests that the VTTC varies across individuals and even for the same person under different circumstances. For VTTC studies, researchers have always been interested, and found empirical evidence, on how the VTTC varies with certain observable variables. In this section, we provide a brief review on some of the most recurrent sources of variation: current trip conditions, personal income and the size and sign of the changes (in time and cost) considered. There may be other important variables explaining the VTTC, but this paper will focus on those mentioned above. For appraisal purposes, current trip conditions and income are particularly important: these are key drivers of the VTTC of a given population, before any survey is conducted. The others are related to the choice context presented in a SC survey (or, if revealed preference methods were used, to the choice context the traveller faced before making his real life choice). In principle, the only certainty is that one should at least try to explain and control for the effects of the choice context attributes.

In the world we live, income is supposed to affect positively the VTTC, while the relationship between current conditions and VTTC is less obvious and not even unidirectional. Current trip conditions, in a simple context, can be understood as the current travel time and current travel cost an individual is facing on the given trip of interest. Ceteris paribus, current time and current cost can affect the VTTC, but also the other way around (e.g. when a person selects his/her current trip conditions based on his/her VTTC). Irrespectively of the direction and sign of the relationships, there is a distribution of the VTTC in a population based on these two variables. In other words, assuming a given trip for each person, there is one VTTC per person in the population. Data and models for VTTC estimation are aimed at picking up this distribution.

Income can be interpreted as personal or household income, and there is evidence showing that the VTTC increases with income (e.g. Small 2012). The impact of current time and current cost is sometimes referred to as “journey length effect” (e.g. Mackie et al. 2003), although the correspondence between current time/cost and distance is not precise. This approximation is, however, practical for appraisal purposes (see e.g. Börjesson and Eliasson 2014). Many empirical applications on the VTTC report the so-called “cost damping” phenomenon, by which the sensitivity towards travel cost decreases as current cost increases (Daly 2010). Analogously, some works also report “time damping”, where time sensitivity decreases as current travel time increases. Across individuals, cost damping and time damping are reasonable especially if we understand that the VTTC can affect the travellers’ current trip conditions (e.g. if a person is choosing an expensive trip in real life, this is likely to be driven by a low sensitivity to cost; *idem* for time). Several empirical works have found that the damping effect on the cost domain is greater than on the time domain, and consequently the VTTC increases with journey length (e.g. Mackie et al. 2003; Börjesson and Eliasson 2014).

The VTTC can also vary for a given individual, depending on the choice context. This creates controversies for appraisal, since ideally the aim is to pick up one unique VTTC per person. For example, the VTTC may be different depending on the sign of the changes considered. Due to diminishing marginal utility, losses would be weighted more than gains (Tversky and Kahneman 1991; De Borger and Fosgerau 2008). Consequently people may value a given saving of 10 min at a lower rate than a loss of 10 min (this is known as loss aversion). Analogously, loss aversion can also be found for the cost attribute. Overall, this translates into a gap between (low) willingness-to-pay measures and (high) willingness-to-accept measures. These effects seem undesirable for appraisal purposes (projects are evaluated with a long-term horizon, where a short-term concept such as loss aversion on a travel choice does not apply) and may be, at least partially, caused by the SC design (e.g. De Borger and Fosgerau 2008; Daly et al. 2014). How to fully remove the choice context effects is unclear, but current consensus is that models should at least control for them (Börjesson and Eliasson 2014). For practical purposes, De Borger and Fosgerau (2008) suggest a formula to obtain one measure of a “reference-free” VTTC.

Also at the level of intra-individual valuation heterogeneity, another source of VTTC heterogeneity (perhaps the most controversial) is the size of the changes considered. Again, this is a choice context effect and may apply in both time and cost domains. Facing two travel options, the difference in travel time between them may be, for example, 5, 10, 15 or 20 min. How the individual values one minute of travel time may vary depending on whether he is considering a bundle of 5,10,15 or 20 min. This is often referred to as “size effects”. Welch and Williams (1997) claimed that small travel time changes (typically below 10 min) should be valued at a lower rate based on SC empirical evidence. The argument is usually supported by signs that individuals may even neglect small time changes (e.g. Mackie et al. 2003). Daly et al. (2014) review this issue, pointing out that although many studies in fact report low valuation of small time changes, the implementation of this finding is controversial and therefore rare. There are certain suspicions that the existing SC methods may not be suitable to accurately estimate this effect due to their artificial nature (Daly et al. 2014). In particular, the difference between two alternatives in a binary survey may have different behavioural implications than the difference between two schemes over time in real world behaviour. Furthermore, other recent studies (Significance 2013; Ojeda-Cabral 2014) recall that there may also be size effects on the cost domain: i.e. VTTC varies with the size of the travel cost change. Börjesson and Eliasson (2014, p.157) conclude that the interpretation and treatment of size effects is “perhaps the outstanding unresolved issue in SC valuation”.

These different ways in which the VTTC has been found to vary empirically have been the object of debate for many years. The empirical work conducted for this paper will shed light on the role of the levels of the SC variables on the estimation of the VTTC, and on how the VTTC varies (e.g. estimation of elasticities).

## Methodology to estimate the VTTC

### Data collection: stated choice experiments

The underlying assumption is that the VTTC can be inferred from individuals’ travel choices where there is a trade-off between travel time (t) and travel cost (c). SC experiments are the most common method to obtain data on this kind of choices. In most European national studies for the VTTC, a binary choice setting is employed. The simplicity of such setting makes it very useful for the purposes of this paper. Each traveller is asked to choose between two travel options: the *fast and expensive* option and the *slow and cheap* option. For ease of exposition, the subscript 1 will always refer to the slow and cheap option, such that: t_1_ > t_2_ and c_1_ < c_2_; in actual surveys, the order of these is obviously randomized across choices. In each choice scenario, there is always a difference in travel time (∆t) and a difference in travel cost (∆c) between the two alternatives. The ratio ∆c/∆t constitutes the BVTTC, an implicit “price of travel time”:

1 $$BVTTC = \frac{{ - (c_{2} - c_{1} )}}{{(t_{2} - t_{1} )}} = - \frac{\Delta c}{\Delta t}$$

In essence, the respondent choosing the fast and expensive (slow and cheap) option would reveal a VTTC higher (lower) than the BVTTC. The BVTTC acts as a valuation threshold. When researchers design a SC experiment, there is always an expectation regarding the range in which the true VTTC will be. This expectation is normally transformed into some kind of “target VTTC” (e.g. Fosgerau and Börjesson 2015), and SC designs are constructed accordingly to be able to pick up the true VTTC. The distribution of the BVTTC presented in the survey defines the *target VTTC.* The importance of the target VTTC was acknowledged early on by Fowkes and Wardman (1988), Fowkes (1996) and Clark and Toner (1997).

### Estimation: discrete choice models

Discrete choice models are used to analyse the data from SC experiments. These models are grounded in microeconomic theory. For many years, the random utility (RU) model has been commonly used (including all European VTTC studies up to 2007; see e.g. Daly et al. 2014). The RU model assumes that individuals choose between two travel options (*i* = 1,2) to maximise their utility. Each travel option *i* is assumed to provide the individual with certain level of utility. The utility (*U*_*i*_) is a function with an observable component and an error (unobservable) component. The error terms in these RU models have typically been incorporated in an additive way, although this is not a requirement:

2 $$\left\{ {\begin{array}{*{20}c} {U_{1} = \beta_{t} *t_{1} + \beta_{c} *c_{1 } + \varepsilon_{1} } \\ {U_{2} = \beta_{t} *t_{2} + \beta_{c} *c_{2} + \varepsilon_{2} } \\ \end{array} } \right.$$

where β_c_ and β_t_ are parameters to be estimated. In this basic setting, they represent the marginal utilities of travel cost and travel time respectively. The VTTC is defined as the marginal rate of substitution between travel time and travel cost, equal to:

3 $$VTTC = \frac{{\beta_{t} }}{{\beta_{c} }}$$

More recently, the random valuation^2^ (RV) model has been used for the last VTTC studies in Denmark, Sweden and Norway (Fosgerau et al. 2007b; Börjesson and Eliasson 2014; Ramjerdi et al. 2010). The intuition of the RV model can be easily seen if the deterministic terms in Eq. (2) are re-arranged (see e.g. Fosgerau et al. 2007a). If the data is re-ordered such that option 1 is always the slow and cheap option, then:

4 $$\left\{ {\begin{array}{ll} {V_{1} = BVTTC = \frac{{ - \left( {c_{2} - c_{1} } \right)}}{{\left( {t_{2} - t_{1} } \right)}}} \\ {V_{2} = VTTC = \frac{{\beta_{t} }}{{\beta_{c} }}} \\ \end{array} } \right.$$

It can be seen that when the *fast and expensive* option 2 provides greater utility then the VTTC is greater than the BVTTC, and vice versa. While the RU model assumes that utility is distributed with constant variance (McFadden 1974) the RV model poses the constant variance assumption on the VTTC (Cameron and James 1987). In all mentioned studies using RV model, the error terms were incorporated in a multiplicative way (again noting that this is not an inherent requirement of the RV approach, just as additive is not a requirement for RU), which in practice translates to the specification of the utility functions in logarithms (see Fosgerau and Bierlaire 2009) as follows:

5 $$\left\{ {\begin{array}{ll} {U_{1} = \mu *\ln (BVTTC) + \varepsilon_{1} = \mu *\ln \left( - \frac{\Delta c}{\Delta t}\right) + \varepsilon_{1}^{\prime} } \\ {U_{2} = \mu *\ln \left( {VTTC} \right) + \varepsilon_{2} = \mu *\ln \left(\frac{{\beta_{t} }}{{\beta_{c} }}\right) + \varepsilon_{2}^{\prime} } \\ \end{array} } \right.$$

with

6 $$VTTC = \frac{{\beta_{t} }}{{\beta_{c} }} = e^{{\beta_{0} }}$$

where β_0_ is a parameter to be estimated (the exponential function simply ensures positivity of the argument of the logarithm and does not affect the results), and $\mu$ is a scale parameter to be estimated associated with $\varepsilon_{i}^{\prime}$ (we use $\varepsilon_{i}^{\prime}$ to note that the type-I GEV distributed error terms in Eq. (5) are not necessarily the same than those from Eq. (2) above). Models can be coded to estimate directly VTTC measures (i.e. $e^{{\beta_{0} }}$).

The latest Danish and Swedish national VTTC studies (e.g. Fosgerau et al. 2007b; Börjesson and Eliasson 2014) employed the RV model, arguing that it was superior to RU. In another paper using the UK national VTTC study data, we show that, for several model specifications, the RV model always performs better than the RU counterpart (Ojeda-Cabral et al. 2015). Consequently, we conduct our analysis using also RV models.

#### Observed and unobserved heterogeneity

Additionally, regardless of the approach selected (i.e. RU or RV), observed and unobserved heterogeneity on the VTTC are generally taken into account. This is done extending the definition of the VTTC in (2) or (6). To account for observed heterogeneity, interactions between the *X*_*j*,_ variables of interest (i.e. those likely to affect the VTTC) and the VTTC are introduced. These interaction terms can modify the VTTC directly. To account for unobserved (or random) heterogeneity, a random parameter *u* is introduced in the VTTC. For example, *u* can be assumed to follow a normal distribution: *u* ~ N(0, σ^2^). In that case, it can be seen straightforwardly that the VTTC follows a lognormal distribution across individuals. The lognormal distribution has the advantage of restricting the VTTC to positive values and has been proven to be convenient in several VTTC applications (see e.g. Börjesson and Eliasson 2014). If these modifications of the VTTC are introduced, the VTTC could be extended as follows:

7 $$VTTC = e^{{\beta_{0} + \beta_{{X_{j} }}^{\prime} X_{j} + u}}$$

where $\beta_{{X_{j} }}^{\prime}$ is a set of parameters to be estimated.

The mean VTTC, accounting for observed and random heterogeneity, can be calculated as:

8 $$E(VTTC) = e^{{(\beta_{0} + \beta_{{X_{j} }}^{\prime} X_{j} )}} e^{{(\frac{{\sigma^{2} }}{2})}}$$

Under simple cost-time settings in SC experiments, the RV approach (in logarithms) and this particular way of introducing observed and unobserved heterogeneity can be regarded as part of the state-of-the-art in model estimation for the VTTC (see Börjesson and Eliasson 2014).

## Dataset

The dataset used in this paper was collected for the national VTTC study in 1994 by Accent and Hague Consulting Group (ACHG 1996). It contains 12,705 choice observations^3^ from car travellers for commute and other (i.e. non-business) purposes.

**Fig. 2:**
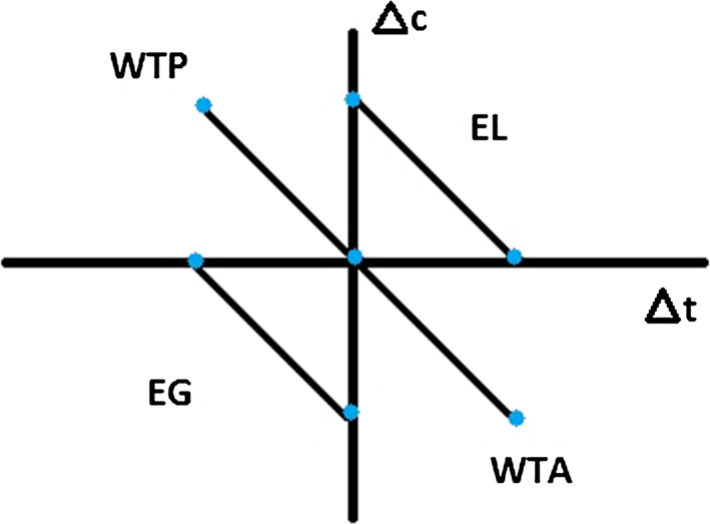
Four types of choices present in most European VTTC studies

The respondents were recruited while travelling and information about a recent trip was collected. This trip, defined by current travel time (*T*) and current travel cost (*C*), is used as the reference trip throughout the survey. The participants were then presented with eight choice scenarios, each with two travel options (*i* = 1,2) varying in travel cost (c_i_) and travel time (t_i_) with levels designed around the reference trip, where Δt_i_ = t_i_−*T* and Δc_i_ = c_i_−*C*. Travellers were asked to choose their preferred travel alternative. This was a forced choice, with no option not to travel. With this basic setup, the SC design of the experiment has some interesting properties. Under each scenario, one of each Δt_i_ and Δc_i_ are set to zero, so travellers are always comparing a given change in time (Δt) against a given change in cost (Δc). In other words, one of the options always contained *T* and one of the options always contained *C*. Those pairwise comparisons are classified into four “types”, based on the four quadrants of an indifference curve map (see Fig. 2 below). The point where the axes intersect represents the current trip of the individuals, i.e. Δt = 0, Δc = 0, from which changes in time and cost are considered:

1. Δt < 0, Δc > 0 (*current journey* vs. faster but more expensive option; willingness to pay or WTP)
2. Δt < 0, Δc < 0 (faster option vs. cheaper option; equivalent gain or EG)
3. Δt > 0, Δc > 0 (slower option vs. more expensive option; equivalent loss or EL)
4. Δt > 0, Δc < 0 (*current journey* vs. slower but cheaper option; willingness to accept or WTA).

The lines linked by dots are a representative example of the slope of the indifference curve that the design tries to capture in each quadrant. In practice, these slopes do not need to be equal for all quadrants; it is theoretically expected (within a Hicksian preference setting) and empirically found that the following relationship holds for the slope: WTP < min(EL, EG) < max(EL, EG) < WTA (see e.g. De Borger and Fosgerau 2008).

Each choice scenario contains an implicit BVTTC. The design includes eight different BVTTC (pence/minute), derived from the different combinations of (Δt, Δc). The values used in the UK experiment for the BVTTC, as well as the time and cost differences making up the BVTTC, are shown in the Table 1 below. The eight BVTTC included were considered to cover a realistic and sufficient range of VTTC at the time of the study, although recent studies (e.g. Börjesson et al. 2012) have suggested that a much larger range of BVTTC is required to identify the true mean VTTC over a sample of choices.

**Table 1:** SC design attribute levels

Design variable	Values used for the SC experiment
Δt (minutes)	−20, −15, −10, −5, −3, +5, +10, +15, +20
Δc (pence)	−300, −250, −225, −150, −140, −125, −105, −100, −75, −70, −50, −35, −30, −25, −20, −15, −10, −5, 5, 10, 15, 20, 25, 30, 35, 50, 70, 75, 100, 105,125, 140, 150, 225, 250, 300
Boundary VTTC (pence/minute)	1, 2, 3.5, 5, 7, 10, 15, 25

In addition to completing the SC tasks, respondents were asked for information on socio-economic characteristics (e.g. income). The work on this data by ITS at the University of Leeds (Mackie et al. 2003) led to the establishment of the current VTTC values officially employed in the UK for appraisal. The analysis carried out in this paper also aims at providing new valuable insights into the results obtained a decade ago, through a focus on the role of the SC design variables.

## Empirical work

The *valuation threshold* is the key element of a SC experiment to estimate individuals’ valuation if simple time-money trade-offs are employed in the experiment. In the context of the VTTC, this is the BVTTC. A key question is whether it is possible that the BVTTC plays a role on the estimation of the set of VTTC. This is not saying that the survey influences preferences, but that, if preferences (and hence VTTC) are different in different settings, then by focussing the survey on specific settings, our sample level results will be affected accordingly. Fosgerau and Börjesson (2015) analyses the impact of the BVTTC on the estimation of the mean VTTC if basic Random Utility models, with no covariates, are employed. Fosgerau and Börjesson “manipulate” (as they claim) a stated choice survey in order to analyse certain impacts of a design variable, in particular the BVTTC. Their work shows empirically that basing the design of the SC experiment on some target VTTC, defined by the distribution of the BVTTC, will bias the estimated VTTC towards the target (if the VTTC is distributed across the sample). In particular, the “bias” is claimed to be related to model misspecification when a simple Random Utility model (e.g. Equation 2 of this paper) is used. Their conclusions simply require that the underlying VTTC in the population vary between individuals (heterogeneity), something which is typically observed in most applications and is therefore acceptable (Fosgerau and Börjesson 2015).

However, much more can be explored. Building upon Fosgerau and Börjesson’s work, our paper will: (a) test different modelling specifications in line with the state-of-the-art (based on the RV model described above^4^); (b) analyse the impact of BVTTC on the estimation of VTTC heterogeneity, i.e. not only on the mean VTTC; and c) test, in a similar fashion, the impact of the components of the BVTTC, namely the change in travel time (Δt) and the change in travel cost (Δc). We assume that estimates are able to reflect preferences, and consequently interpret any differences in estimates as differences in preferences. This empirical work is an attempt to increase our understanding of the role of the design variables in VTTC studies and how inferred preferences can be different depending on the design setting.

### Partial analysis of the data

The series of empirical exercises conducted to investigate our research questions may be regarded as *partial data analysis*: the term “manipulation” employed by Fosgerau and Börjesson (2015) might be misleading for our paper. We conduct three exercises, where in each the dataset of travel choices is split into several sub-samples based on some variable of interest. In this work, three variables of interest (one per exercise) are investigated: the BVTTC, Δc and Δt. The levels of these three variables were selected by the researchers when constructing the SC experiment (Accent Marketing and Research and Hague Consulting Group 1996). In each exercise, the same model will be estimated on each sub-sample, as well as on the full dataset. The use of this is not to try to show the inabilities of an inferior (RU) model, but to investigate patterns on the estimation results across sub-samples, in relation to the VTTC and its covariates.

In order to divide the data, Fosgerau and Börjesson (2015) employ the quartiles of the BVTTC distribution. This gives four sub-datasets of similar size, each with an exclusive range of BVTTC. Afterwards, the same discrete choice model is estimated on the four sub-datasets. In our three exercises, we will make use of the same state-of-the-art model specifications (a logarithmic RV model), in line with Eqs. (5) to (8). The set of *X* covariates employed for the definition of the VTTC (Eq. 8) is the following:

9 $$\beta_{{X_{j} }}^{\prime} X_{j} = \beta_{BC} \ln \left( {\frac{C}{{C_{0} }}} \right) + \beta_{BT} \ln \left( {\frac{T}{{T_{0} }}} \right) + \beta_{I} \ln \left( {\frac{I}{{I_{0} }}} \right) + \beta_{\Delta t} \ln \left( {\frac{\Delta t}{{\Delta t_{0} }}} \right)$$

where *C* = Current travel cost, *C*_0_ = Reference level of current travel cost (e.g. sample average = 550 pence), *T* = Current travel time, *T*_0_ = Reference level of current travel time (e.g. sample average = 70 min), *I* = Income of the individual, *I*_0_ = Reference level of income (e.g. sample average = £25,000), ∆*t* = Change in travel time, ∆*t*_0_ = Reference level of change in travel time (e.g. sample average = 7.7 min).

Additionally, the base estimate for the VTTC in Eq. (8), represented by $e^{{\beta_{0} }}$, will be estimated separately for each of the four types of choices (i.e. quadrants) present in the data. Altogether, this VTTC definition allows us to capture all sources of variation mentioned in “Evidence on variation in the VTTC” section, together with random heterogeneity. Other sources of systematic taste variations (e.g. age, gender or trip purpose) were tested but found not significant; only main effects were kept in our models, which is also common practice for models that are then used for policy (e.g. Mackie et al. 2003). Note that, in a given RV model (and with data using the design explained above), it is only possible to identify how the VTTC varies with either Δt or Δc. The reason is that the BVTTC, Δc and Δt are multicollinear and the BVTTC is already part of the model. We have selected Δt as a source of variation, since this is typically a key factor in VTTC studies and also to comply with current practice.

Alongside the VTTC measures by quadrant (four choice quadrants of the indifference map), the geometric average of the four will also be provided as a measure of the VTTC in the sample:

10 $${\rm VTTC} = ({\rm VTTC}_{\rm WTP} * {\rm VTTC}_{\rm WTA} * {\rm VTTC}_{\rm EL} * {\rm VTTC}_{\rm EG} )^{1/4}$$

This would be an approximation to what De Borger and Fosgerau (2008) suggest as “reference-free” VTTC, for this sort of choice settings, through a theoretical derivation. This approximation is sufficient for the purposes of this paper, and is certainly better than a mean VTTC derived from a model that did not account for the differences across quadrants (De Borger and Fosgerau 2008). In any case, the use of the geometric mean does not affect the conclusions of this work, since we would get the same insights by simply looking at the specific VTTC by quadrants.

#### Exercise 1-data split by BVTTC

In the first exercise, the data is split into four based on the quartiles of the BVTTC distribution. Two models are estimated on each sub-sample: with and without random heterogeneity.

For a more complete analysis, the models are also estimated for the full sample. The models on the full sample will also be comparable with the models from “Exercise 2-data split by Δt” and “Exercise 3-data split by Δc” sections that will be presented later on. All models have been estimated using Biogeme (Bierlaire 2003).

The estimation results are shown in Tables 2 and 3, where the former is for a Multinomial Logit model and the latter for a Mixed Logit model. In all our models, the panel nature of the data was taken into account in the calculation of the robust standard errors. In the random coefficients models, the random heterogeneity was specified to be across respondents, maintaining homogeneity in sensitivities across the choices for the same person. We will look at differences in the VTTC and in the estimation of observed and random heterogeneity. On each table, each column defines a sub-sample, showing the available range of the design variable for which the model is estimated.

**Table 2:** Data split by BVTTC-Estimation results (Standard errors in parentheses)

4 Sub-Samples, each with different BVTTC range					
	(1,2)	(2,5)	(5,10)	(10,25)	ALL
Mean VTTC (censored max BVTTC*1.25)*	1.09	3.52	4.8	6.2	4.89
Median VTTC (p/min)	2.13 (0.61)	4.27 (0.204)	5.2 (1.21)	4.68 (1.74)	3.13 (0.14)
VTTC_EG_	1.88 (0.118)	4.33 (0.163)	5.33 (0.348)	7.52 (1.71)	3.67 (0.244)
VTTC_EL_	1.91 (0.14)	3.82 (0.105)	4.28 (0.397)	3.17 (1.1)	2.07 (0.157)
VTTC_WTA_	4.85 (0.618)	6.03 (0.247)	10.1 (0.477)	10.5 (1.4)	9.97 (0.701)
VTTC_WTP_	1.19 (0.0866)	3.33 (0.117)	3.17 (0.484)	1.91 (0.821)	1.27 (0.105)
β_BC_	0.157 (0.052)	0.183 (0.03)	0.21 (0.052)	0.41 (0.15)	0.41 (0.06)
β_∆T_	NA	NA	NA	NA	0.711 (0.0766)
β_I_	0.28 (0.0484)	0.141 (0.0239)	0.193 (0.0437)	0.387 (0.119)	0.472 (0.0566)
β_BT_	−0.025 (0.07)	−0.098 (0.033)	−0.14 (0.054)	−0.188 (0.134)	−0.406 (0.0825)
μ	1.32 (0.116)	2.74 (0.211)	1.72 (0.217)	1.06 (0.2)	0.781 (0.0252)
obs	3382	3328	3100	2420	12230
Indiv.	1776	1769	1673	1399	1804
LL	−2013.35	−1944.49	−1788.274	−935.679	−6708.528
adj. ρ2	0.138	0.154	0.164	0.437	0.208

* Standard errors could not be calculated for the mean VTTC when it required simulation and censoring

**Table 3:** Data split by BVTTC-Estimation results (Standard errors in parentheses)—Random Heterogeneity

	(1,2)	(2,5)	(5,10)	(10,25)	ALL
Mean VTTC	2.89 (0.25)	4.61 (0.13)	8.62 (1.43)	10.89 (1.19)	7.04 (0.5)
β_BC_	0.102 (0.04)	0.22 (0.0324)	0.397 (0.109)	0.436 (0.13)	0.337 (0.057)
β_∆T_	NA	NA	NA	NA	0.94 (0.058)
β_I_	0.24 (0.04)	0.144 (0.025)	0.321 (0.09)	0.31 (0.09)	0.396 (0.05)
β_BT_	−0.023 (0.06)	−0.06 (0.035)	−0.41 (0.124)	−0.016 (0.12)	−0.43 (0.076)
μ	1.95 (0.153)	3.67 (0.3)	1.31 (0.264)	1.96 (0.331)	1.02 (0.0321)
β0	0.768 (0.049)	1.41 (0.0222)	1.47 (0.142)	1.49 (0.233)	1.17 (0.0535)
σ	0.768 (0.07)	0.485 (0.04)	1.17 (0.239)	1.34 (0.22)	1.25 (0.0492)
obs	3382	3328	3100	2420	12230
Indiv.	1776	1769	1673	1399	1804
LL	−2104.241	−1962.843	−1872.23	−938.9	−6636.473
adj. ρ2	0.1	0.147	0.126	0.437	0.216

The first row shows an estimate of the mean VTTC, at the sample average of the covariates. In Table 3, where a lognormal distribution is assumed for the VTTC, the mean is easily calculated using Eq. (8) (Fosgerau et al. 2007a, b). In Table 2, where the only random term in the model is the logistic error, the direct model estimates of the VTTC will correspond to the median instead of the mean of the (logistic) distribution.^5^ The derivation of the mean requires simulation and censoring of the distribution. The mean VTTC can be calculated taking the logistic distribution of the error (the difference of two type-I GEV distributed error terms follows a logistic distribution) into account as follows (Ojeda-Cabral et al. 2015):

11 $${\rm Mean} \,{\rm VTTC} = \exp \left[\ln \left( {e^{{\beta_{0} }} } \right) + \frac{1}{\mu }\left( {\varepsilon_{1}^{\prime} - \varepsilon_{2}^{\prime} } \right)\right]$$

where all parameters correspond to the model described in Eqs. (5) and (6) in “Estimation: discrete choice models” section, and hence where $e^{{\beta_{0} }}$ is equal to the median VTTC. Calculation requires simulation for the logistic distributions. Additionally, since those distributions are unbounded, it is necessary to make an assumption for the VTTC values which the data does not support, given mainly by the range of BVTTC (see Börjesson et al. 2012). We use the approach followed in the Danish VTTC study (Fosgerau et al. 2007b) and censor the simulated VTTC distribution, restricting it to be close to the BVTTC range. For this, we use a censor value just above the upper limit of the BVTTC range of the sample (1.25 * upper limit).

Rows below the mean VTTC measure provide the estimates of all coefficients for each model, including specific VTTC measures by choice context (WTA, WTP, EL and EG). All VTTC measures are provided in pence per minutes. The standard errors of all estimates are included in brackets. The last four rows report the number of observations and individuals on each sub-sample, together with the Log-Likelihood and adjusted rho-squared as model fit indicators.

Overall, coefficients in all models are estimated with the expected sign based on theory and previous evidence on this dataset (e.g. Mackie et al. 2003; Ojeda-Cabral 2014), and all levels of significance are reasonable. The restricted sub-samples caused, in some cases, identification or correlation problems for estimation due to lack of variability of some variables. As a result, the models had to be simplified for those samples as will be explained below. For ease of exposition, the results will be interpreted in several steps as follows.

##### VTTC estimation (mean/median)

We observe a tendency of the VTTC towards the BVTTC, in line with the exercise using a basic Random Utility model carried out by Fosgerau and Börjesson (2015). Note that Fosgerau and Börjesson (2015)’s exercise was also replicated on our dataset, finding the same tendency when using a RU model.^6^ Here we show that the mean VTTC significantly increases as the BVTTC increases, also with a RV model (only the difference between the values in columns 3 and 4 is not significant). The differences are also of a relevant size that would be meaningful if these VTTC were to be recommended for appraisal (e.g. Department for Transport 2013). This is an unexpected and important result, as it shows that even a model that is regarded as a good descriptor of this kind of the data would pick up a positive relationship between VTTC and BVTTC. The estimated preferences seem to reveal a valuation that is proportional to the valuation threshold offered. Additionally, the increases in VTTC with BVTTC seem to be especially driven by WTA and EL choice contexts.

The VTTC measures by quadrant (WTA, WTP, etc.) show a large gap between WTA and WTP contexts, in line with findings in the literature (e.g. De Borger and Fosgerau 2008), but also between EL and EG.

How precise are the VTTC estimates when the SC design setting is limited? Large standard errors are normal due to sample size reduction, but there are differences across sub-samples. Most coefficients are much more precise in the second sub-dataset (BVTTC = (2, 5]). The scale of the models (μ) is also greater in the second sub-sample. Interestingly, the estimated median VTTC in all sub-datasets falls into this range, between 2 and 5 pence/minute. Our interpretation is that offering BVTTC levels that are close to the true VTTC increases the precision of the estimation. This could also be regarded as a positive feature of the RV approach, which would be able to approximately recover the underlying median VTTC in the sample regardless of the BVTTC range presented.

It is interesting to compare our estimates with the official work conducted on this data by Mackie et al. (2003), which led to the national appraisal values that are still used in practice. For a fair comparison,^7^ in Ojeda-Cabral (Ojeda-Cabral et al. 2015) the replication of the official model employed by Mackie et al. (2003) led to a VTTC of 5.19 pence/minute. The mean VTTC estimated from our best model on the full sample (Table 3) is almost 2 pence/minute higher, which would be determinant in the appraisal of projects (Department for Transport 2013). By sub-samples, the 5.19 estimate would be closer to the value from the sample with BVTTC between 2 and 5.

##### Random heterogeneity

The inclusion of random heterogeneity on top of the models from Table 2, through the assumption of a lognormal distribution for the VTTC, caused problems in estimation, but only when not all data was used. If all data is used, the same full model was estimated and the inclusion of random heterogeneity improved model fit considerably. However, when only a sub-sample is used, the standard deviation (*σ*) of the underlying normal distribution assumed for *u* (Eq. 8) was almost always not significant. These models are not reported here but are available on request. To be able to estimate models that made use of the lognormal assumption, several options were tested. A solution was to estimate only one VTTC (not specific by quadrants) and to omit size effects (β_∆T_ = 0). These problems suggest potential confounding issues between choice context effects and the assumption of a lognormal distribution that are discussed below.

Crucially, the introduction of random heterogeneity allows us to observe that the tendency of the mean VTTC towards the BVTTC is statistically significant, even if the RV model used is believed to represent a more realistic choice process for the data analysed and hence fits the data better than a RU model (Fosgerau and Borjesson, Fosgerau and Börjesson 2015). The failure to estimate the full model on sub-samples can be interpreted in two ways. First, as a sign that a reasonably wide range of BVTTC is necessary in order to capture a distribution (whether lognormal or other) of the VTTC, while picking up choice context effects simultaneously (size effects and quadrants effect). On the other hand, another hypothesis might be that the random heterogeneity that we observe in the model on the full sample is actually an artefact caused by not fully capturing those choice context effects, including the observed variation with the BVTTC across sub-samples. While support for the first hypothesis can be found in the existing literature (e.g. Börjesson and Eliasson 2014), this does not mean the second hypothesis might not hold (it would be an interesting objective for further research, e.g. with the use of simulated data).

##### Covariates

Across the different sub-samples, there are no major variations in the magnitude of the estimates. Heterogeneity across individuals is found in all cases and estimation is relatively consistent. Heterogeneity within individuals due to the size of time changes (β_∆T_) could not be estimated in some sub-samples due to the reduced BVTTC range, and for comparison purposes we set β_∆T_ = 0 in all sub-samples. In general, a given covariate always takes the same sign but again the standard errors of coefficients are lower for the sub-sample with BVTTC between 2 and 5. Hence, the estimation of covariates is also more precise in this range of the BVTTC.

The *income elasticity* (β_I_) is always highly significant around 0.2–0.4, showing that VTTC is higher the higher the income of the individual. At the same time, the *current time elasticity* (β_BT_) is, interestingly, generally about the same magnitude than the *current cost elasticity* (β_BC_) but with the opposite sign (i.e. cost damping and time damping effects approximately compensate each other). In a context (car travel) where current time and cost are positively correlated and directly related to journey length, this suggests a lack of journey length effect (opposite to Mackie et al. 2003, who reported a VTTC that increased with journey length due to cost damping, because their model omitted β_BT_). It must be noted that these elasticities are cross-sectional, and do not necessarily reflect how VTTC would evolve over time with income and journey length. The estimated values are in line with other works (e.g. Mackie et al. 2003; Fosgerau et al. 2007a, b).

With respect to the influence of the size of the changes, the estimate of β_∆T_ in the full sample indicates that the VTTC increases with the size of the time changes, in line with most empirical evidence (e.g. De Borger and Fosgerau 2008; Stathopoulos and Hess 2012). However, β_∆T_ was non-significant in some sub-samples, and non-identifiable in others, which show that the estimation of size effects suffers from some kind of dependency on the range of BVTTC in the sample.

#### Exercise 2-data split by Δt

In exercises 2 and 3, the data will be analysed again partially, but now in relation to the two components of the BVTTC separately. In the case of Δt, there are only five levels for this variable in absolute terms (3, 5, 10, 15 and 20). Hence, a more interesting analysis is to subdivide the data into five sub-samples (one for each level of Δt), rather than using ranges by quartiles. It is worth pointing out that this is possible with a RV model, but not with a RU model (in a RU model, estimation requires variability in ∆c and ∆t). One advantage of the RV approach over RU is that, as long as there is variation in the BVTTC, the model can be estimated even if there is no variation in one of its two components (i.e. ∆c or ∆t). Results are shown in Table 4 below.

**Table 4:** Data split by Δt-Estimation results (Standard errors in parentheses)

5 Sub-Samples, split by ∆t						
∆t	3	5	10	15	20	ALL
Mean VTTC (cens. 30p/min)	2.93	4.85	5.23	6.32	6.69	5.14
Median VTTC	1 (0.27)	2.89 (0.39)	3.34 (0.31)	4.54 (1.04)	7.6 (2.41)	3.37 (0.25)
VTTC_EG_	1.83 (0.37)	3.83 (0.42)	4.66 (0.5)	6.79 (1.68)	6.67 (2.12)	3.38 (0.214)
VTTC_EL_	NA	1.63 (0.22)	2.61 (0.22)	2.46 (0.43)	4.55 (1.14)	2.61 (0.176)
VTTC_WTA_	NA	8.38 (0.9)	9.2 (0.858)	17.6 (4.79)	22.6 (6.45)	11.3 (0.757)
VTTC_WTP_	0.55 (0.22)	1.33 (0.19)	1.11 (0.15)	1.44 (0.633)	4.84 (1.32)	1.29 (0.1)
β_BC_	0.49 (0.14)	0.37 (0.08)	0.35 (0.09)	0.41 (0.19)	1.38 (0.3)	0.43 (0.06)
β_I_	0.52 (0.15)	0.39 (0.07)	0.5 (0.07)	0.42 (0.14)	0.49 (0.17)	0.45 (0.05)
β_BT_	−0.23 (0.19)	−0.27 (0.1)	−0.41 (0.11)	−0.35 (0.24)	−1.38 (0.4)	−0.2 (0.075)
μ	0.65 (0.09)	0.84 (0.05)	0.83 (0.04)	1.16 (0.19)	0.77 (0.1)	0.82 (0.02)
obs	2142	4485	4007	545	1051	12230
Indiv.	1100	1369	1495	228	286	1804
LL	−1067.982	−2419.609	−2277.025	−253.793	−628.896	−6777.606
adj. ρ2	0.277	0.219	0.177	0.307	0.126	0.2

The results show that the VTTC increases consistently with the level of Δt, in line with existing literature (e.g. Mackie et al. 2003; Cantillo et al. 2006). However, most of the differences are not statistically significant, and only the VTTC when Δt = 3 is significantly lower than the rest at the 90 % confidence level. This could be because we are only observing the median, but additional models (available on request), show that the VTTC is also significantly higher when Δt = 20. More importantly, what this exercise shows is the identification of a type of choice context which highly drives the VTTC up: WTA scenarios with increases in journey time of 15 and 20 min. People reveal a very high VTTC when asked about extending their typical journey by 15 or 20 min. In other words, they would ask for a very high monetary compensation in these contexts. Scale, across sub-samples, is lower for Δt = 3 and Δt = 20, indicating more uncertainty in those choices. This could reflect that choices are more difficult when the changes are too small or too large.

However, it must be noted that the sample size is much larger for levels Δt = 5 and Δt = 10. Only a small part of the respondents observed the higher levels and other effects (e.g. personal/trip characteristics) may be at play in determining the higher values when Δt = 15 and Δt = 20. To control for this, VTTC estimates are always calculated for the same level of income and trip characteristics.

The estimated covariates suggest that income and journey length effects are similar for any level of time changes considered: income elasticity is around 0.4 and the effects of current time and current cost on VTTC approximately compensate each other so there is no journey length effect overall, in line with exercise 1. Interestingly, β_BC_ and β_BT_ are both much greater when Δt = 20, and of the same exact magnitude on average but opposite sign. The message is that the effect of a trip characteristic may be influenced by the choice scenario, or in other words, some VTTC elasticities may not be constant. Also, as in exercise 1, the fact that the effects of *C* and *T* always compensate each other in every sub-sample provides strong evidence that journey length effects do not exist in this dataset, regardless of the levels of time changes.

#### Exercise 3-data split by Δc

The data is split based on the quartiles of Δc for the last exercise. The results are shown in Table 5. The results are close to those observed for exercise 1, where the data was split by BVTTC (which makes sense, since BVTTC = Δc/Δt).

**Table 5:** Data split by Δc-Estimation results (Standard errors in parentheses)

4 Sub-Samples, split by ∆c					
∆c range	(5–15)	(16–35)	(35–75)	(75–300)	ALL
Mean VTTC (cens. 30p/min)	3.92	5.4	6.82	6.37	4.89
Median VTTC	2.07 (0.85)	3.64 (0.48)	5.2 (1.06)	4.83 (0.87)	3.13 (0.14)
VTTC_EG_	1.72 (0.165)	3.86 (0.278)	5.84 (0.456)	6.78 (0.905)	3.67 (0.244)
VTTC_EL_	1.77 (0.179)	2.99 (0.231)	3.83 (0.27)	3.5 (0.597)	2.07 (0.157)
VTTC_WTA_	5.45 (0.951)	7.39 (0.844)	9.38 (0.585)	12 (0.874)	9.97 (0.701)
VTTC_WTP_	1.11 (0.111)	2.06 (0.194)	3.5 (0.317)	1.9 (0.482)	1.27 (0.105)
β_BC_	0.164 (0.062)	0.374 (0.075)	0.267 (0.0618)	0.418 (0.113)	0.406 (0.0635)
β_∆T_	NA	NA	NA	NA	0.711 (0.0766)
β_I_	0.304 (0.06)	0.381 (0.0631)	0.246 (0.0473)	0.32 (0.0824)	0.472 (0.0566)
β_BT_	−0.1 (0.086)	−0.35 (0.083)	−0.25 (0.062)	−0.372 (0.109)	−0.406 (0.0825)
μ	1.09 (0.0853)	1.04 (0.0916)	1.56 (0.132)	1.11 (0.12)	0.781 (0.0252)
obs	3593	3385	2832	2420	12230
Indiv.	1523	1703	1516	1145	1804
LL	−1989.3	−2059.113	−1464.179	−1168.29	−6708.528
adj. ρ2	0.198	0.119	0.25	0.299	0.208

The mean and median VTTC varies slightly among sub-samples in a similar way than in exercise 1. The VTTC seems to increase with the level of Δc, but the differences (for the medians) are not statistically significant. It may be expected to find a positive tendency if the lognormal assumption was introduced,^8^ in line with exercise 1, due to the correlation between BVTTC and Δc. The sub-sample where Δc is between 35 and 75 pence presents higher scale and slightly more precise coefficients. As in “Exercise 1-data split by BVTTC” section, this could happen because this sub-sample mostly comprises values of the BVTTC which are close to the underlying VTTC of respondents.

Heterogeneity within individuals, i.e. VTTC variation with Δt, could not be estimated when the range of Δc was reduced. Regarding heterogeneity across individuals, the estimates of the covariates vary slightly across sub-samples, but again the same patterns are observed. Income effect is, again, the most stable across sub-samples and β_BC_ and β_BT_ always compensate each other reflecting lack of journey length effect.

## Discussion and conclusions

This paper has investigated whether and how preferences are different under different SC design settings. More precisely, we studied the role of the variables used in the SC experiment on the estimation of the set of VTTC (i.e. mean and covariates). Our aim has been to increase our understanding of the reality of the distribution of the VTTC in a given population. For this, we used partial data analysis on data collected with a simple VTTC experiment, where the design variables are time changes (Δt), cost changes (Δc) and the implied boundary VTTC (BVTTC = Δc/Δt). The motivation was the finding that VTTC tends to the BVTTC that Fosgerau and Borjesson (Fosgerau and Börjesson 2015) report, finding attributed to a model misspecification. We extended the analysis by using more sophisticated models, observing not only the mean VTTC but its sources of heterogeneity, and by looking also at other design variables.

The main conclusion is that key model estimates (i.e. VTTC and covariates) are different in different design settings (where the settings are defined by the levels of the design variables). In other words, preferences do vary with the SC setting. Hence, if model estimates are different in different settings, then by focusing the survey on specific settings, our sample level results will be affected accordingly. We can only reveal preferences for the particular SC setting that we use, and our results show that the selection of a particular setting can be crucial. SC designs should be constructed bearing this in mind.

The results can be summarized as follows. First, the VTTC varies with the levels of the design variables. Surprisingly, the BVTTC does have a direct positive impact on the estimated VTTC even if a model is employed that is commonly used and known to describe accurately this type of data. The size of cost changes, largely correlated with BVTTC, has a similar direct impact on the VTTC. The size of time changes affects positively the VTTC, but this effect was not identifiable or not significant when the range of BVTTC was reduced (i.e. under some SC design settings). Secondly, more precise estimates (or preferences) are obtained the closer the levels of BVTTC are to the underlying VTTC. This applies to all coefficients.

Third, the introduction of random heterogeneity (through the assumption of lognormal distribution for the VTTC) showed that a wide range of BVTTC values is needed to capture the whole VTTC distribution (e.g.Börjesson et al. 2012) while picking up other effects. Researchers then face a trade-off when designing the experiment: a focus around the expected VTTC (if known) could bring more precise estimates, but covering a reasonably wide range can allow capturing a potential VTTC distribution. However, we cannot discard the hypothesis that the random heterogeneity that we observe in the model on the full sample may actually be an artefact caused by not fully capturing choice context effects, including the observed variation with the BVTTC across sub-samples. The test of this confounding hypothesis, e.g. with the use of simulated data, is suggested as an interesting objective for further research.

Fourth, inter-personal variation of the VTTC is reasonably consistent regardless of the SC design setting. The estimates of the covariates suggest, quite consistently, that: i) income effects are always present in a relatively similar magnitude, and ii) journey length effects do not seem to exist in the data explored, since the VTTC increases with current cost but decreases with current time always in a similar magnitude, compensating each other. Finally, some particular SC design settings infer extremely low/high VTTC estimates. The gap between WTA and WTP is very large, in some cases of a ratio of 5. In general, all WTA scenarios are associated with higher VTTC, but in particular those WTA scenarios where the size of the time changes is 15 or 20 min.

It is known that valuation studies should approach carefully the selection of valuation thresholds for SC designs, and more generally choice contexts. This paper provides new and strong evidence that SC design selection involves decisions that are not innocuous, such as the range and focus of the BVTTC or the inclusion of some WTA scenarios. The good news is that we have also seen how a state-of-the-art model (Random Valuation model), even with a sample based on a limited SC design setting, is able to provide significant and reasonable estimates of the VTTC and its main covariates at the inter-individual level (income and journey length). However, the RV model also provides estimates of the VTTC that tend towards the range of the BVTTC present in the data.

It is not the purpose of this paper to give specific guidelines on how to choose a good SC design. Every market where valuation information is needed is different, and researchers would have to make the design selection that they find more appropriate. Our hope is that this paper raise awareness about the variation of preferences with choice contexts, and hence the importance of selecting the experiment carefully. Preferences are different under different SC design settings, and it is the responsibility of the researcher to decide what set of preferences is wanted.

## Footnotes

1 The researcher decides which variables (e.g. changes in travel time) and which levels of the variables (e.g. 5, 10 and 15 min) to use in a SC experiment to provide respondents with meaningful and realistic choice contexts. Valuation may be different under different contexts.

2 This terminology is used by (Hultkranz et al. 1996) and Ojeda-Cabral et al. (2015).

3 70 observations were removed due a mistake in the design which offered a negative BVTTC in those cases.

4 Note that (Fosgerau and Börjesson 2015) use a different terminology for the RV model described in this paper: they refer to it as ‘random MRS model’.

5 The median will coincide with the mean if the logistic error is not part of the preferences. This, however, is contrary to expectations based on empirical evidence (e.g. Börjesson and Eliasson 2014). If the VTTC follows a distribution and there are not specific random terms to capture it, it can be partially captured by the logistic error.

6 These results are available from the corresponding author on request.

7 Mackie et al. (2003) reports VTTC estimates after weighting was applied using National Travel Survey (NTS) data.

8 This and other modelling exercises have been tested, but did not enrich the analysis and would have extended the length of the paper considerably.
